# Sepsis and the immunometabolic inflammatory response

**DOI:** 10.1038/s44324-025-00095-w

**Published:** 2026-02-02

**Authors:** Samuel N. Paul, Isabell Nessel, Zudin Puthucheary, Siân M. Henson

**Affiliations:** 1 Centre for Translational Medicine and Therapeutics, William Harvey Research Institute, Queen Mary University of London, London, UK; 2 Adult Critical Care Unit, The Royal London Hospital, Barts Health NHS Trust, London, UK

**Keywords:** Biomarkers, Computational biology and bioinformatics, Diseases, Immunology, Medical research

## Abstract

Sepsis is a life-threatening syndrome characterised by dysregulated immunity, inflammation and metabolic disruption. Despite improved care, it remains a major cause of morbidity and mortality, highlighting the need for improved mechanistic insight. Immunometabolism has emerged as a framework for understanding sepsis pathophysiology. Conventional prognostic tools reflect downstream organ injury but not the metabolic states of immune cells. Emerging technologies now enable high-resolution profiling of immunometabolic changes and integrating these approaches may yield metabolic biomarkers capable of tracking immune function and refining diagnostics. This review summarises current knowledge of leukocyte metabolic dysfunction in sepsis and highlights how immunometabolic profiling can inform patient monitoring and advance biomarker-driven precision medicine.

## Introduction

Sepsis is a life-threatening syndrome where a dysregulated host response to infection results in systemic inflammation, immune dysfunction and metabolic disruption. Septic shock is a more severe form, defined clinically by hypotension, vasopressor dependence, and elevated lactate, and carries markedly higher morbidity^1^. Globally, sepsis accounted for nearly one in five deaths in 2017, with children under five comprising almost half of those deaths^2^.

Traditionally, sepsis has been viewed as a biphasic condition. First involving an intense hyperinflammatory response, triggered by pro-inflammatory mediators^3^ and metabolic disturbances in reaction to infection or injury^4^. This is followed by a compensatory immunosuppressive phase, during which regulatory mechanisms act to restrain inflammation, promote tissue repair, and restore physiological balance^5^. However, patients rarely follow this simple sequential pattern and contemporary models of sepsis now recognise that hyperinflammatory and immunosuppressive processes co-exist from the onset of the disease. The progression of sepsis instead follows a distinct pattern of immunometabolic and phenotypic remodelling, where both innate and adaptive immune responses undergo temporally distinct shifts^6^. These changes are not static, with immune cells displaying alterations in phenotype and metabolism across different stages of sepsis, reflecting a continuous interplay between immune activation, suppression and metabolic reprogramming.

Emerging evidence identifies metabolic dysfunction as a central driver of immune dysregulation in sepsis. This review will focus on immunometabolic alterations in leukocytes during sepsis, examining how changes in fuel usage and mitochondrial function shape immune cell phenotype and function. We will also discuss the potential of metabolic biomarkers to monitor immune status, highlighting the role of immunometabolic profiling as a tool for understanding sepsis pathophysiology and guiding precision diagnostics.

## Immunometabolism in Sepsis

**Fig. 1:**
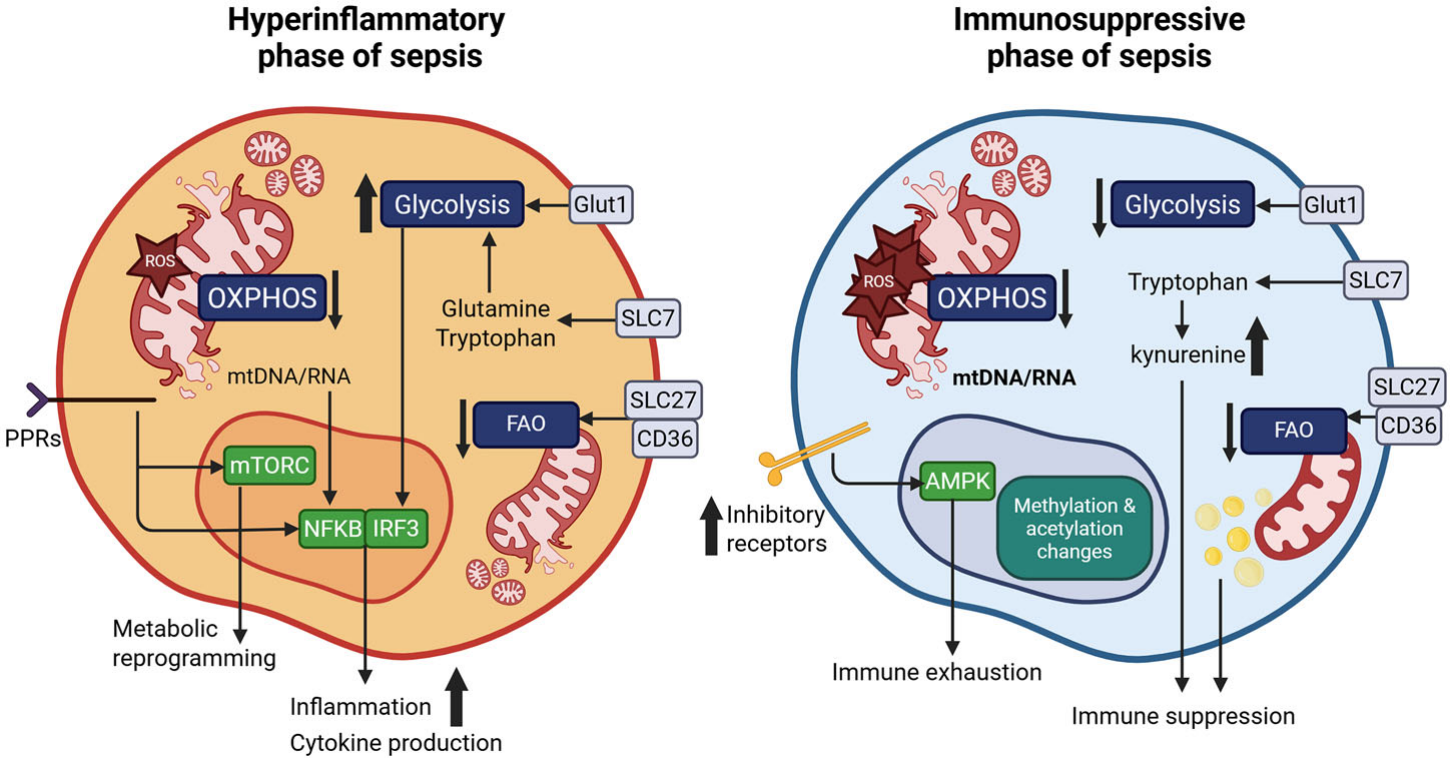
Immunometabolic reprogramming in sepsis. Immune cells in the hyperinflammatory phase of sepsis (left) shift toward aerobic glycolysis and increase amino acid uptake supporting rapid inflammatory activation and effector function. As sepsis progresses, impaired FAO and reduced OXPHOS emerge due to mitochondrial dysfunction and altered nutrient transporter activity. These metabolic defects limit ATP production, promoting transition from hyperinflammation to immune suppression (right), characterised by increased expression of inhibitory receptors driving immune exhaustion. Rising kynurenine levels act as an immune suppressant, together with the accumulation of oxidised lipids which generate immunosuppressive lipid mediators. Collectively, these changes redirect the immune response from effective host defense toward tissue injury and organ dysfunction^103^.

During a systemic infection such as sepsis, the immune system mounts an intense response aimed at eliminating invading pathogens, characterised by rapid cellular activation, proliferation and cytokine synthesis^7^. These processes demand not only increased glucose utilisation through glycolysis but also extensive remodelling of amino acid metabolism to sustain biosynthetic and energetic needs^8^. While this organism-wide hypermetabolic state supports pathogen clearance, it can simultaneously deplete resources needed for disease tolerance, which depends on catabolic processes to preserve tissue structure and function through energy derived from macromolecule breakdown^8^. Conversely, mechanisms designed to suppress excessive inflammation can shift metabolism toward a predominantly catabolic state that prioritises limiting tissue damage. When exaggerated, this can impair pathogen clearance, increase vulnerability to secondary infections, and promote irreversible organ failure^5^. Ultimately, survival depends on maintaining a dynamic equilibrium between hypermetabolic and hypometabolic programs, a balance that becomes profoundly disrupted during sepsis (Fig. 1).

In the hyperinflammatory phase, innate immune cells such as macrophages, neutrophils, and natural killer (NK) cells are rapidly activated through pattern-recognition receptors like toll-like receptors (TLR)^9^. Engagement of TLRs triggers NF-κB and interferon pathways, leading to the production of pro-inflammatory cytokines including TNF-α, IL-1β, and IL-6^9,10^. While essential for pathogen clearance, dysregulated cytokine activity drives vascular leak, hypotension, and edema^1^. These signalling cascades trigger transcriptional programs that upregulate glycolytic enzymes and glucose transporters, resulting in elevated glycolytic flux^11^. In macrophages, glycolytic reprogramming promotes polarisation toward the M1 pro-inflammatory phenotype, supporting cytokine production and antimicrobial activity, while limiting the energy available for M2 anti-inflammatory and reparative functions^12^. This reprogramming is coordinated by signalling pathways such as mammalian target of rapamycin (mTOR), hypoxia-inducible factor 1a (HIF-1α)^13^, and reinforced by TLR4 engagement, glucose transporter 1 (GLUT1) upregulation, along with key glycolytic control points like 6-phosphofructo-2-kinase/fructose-2,6-bisphosphatase 3 (PFKFB3)^14^.

Adaptive immunity is similarly affected. In CD4⁺ T cells, glycolysis promotes differentiation into pro-inflammatory Th1 and Th17 subsets, driving IFN-γ and IL-17 secretion^15^, whereas in CD8⁺ T cells, glycolysis supports granzyme and perforin production fuelling the early effector response^16^. In contrast, regulatory T cells (Tregs), which rely on oxidative phosphorylation (OXPHOS) and fatty acid oxidation (FAO) are suppressed under glycolytic conditions tipping the immune balance toward inflammation^17^. When unchecked, excessive glycolysis contributes to sustained hyperinflammation, oxidative stress and immune cell dysfunction^18^. Persistent activation amplifies inflammatory signalling, exemplified by nuclear translocation of pyruvate kinase isoenzyme M2 (PKM2), which enhances HIF-1α–mediated transcription of glycolytic enzymes and IL-1β^19,20^. These processes drive tissue injury, endothelial dysfunction, and ultimately organ failure. Clinically, this hypermetabolic state is reflected by elevated serum lactate, which reflects both tissue hypoperfusion and heightened glycolytic flux in activated immune cells^21^.

Amino acids provide essential substrates for protein synthesis, nucleotide generation and antioxidant defense, supporting both immune cell expansion and effector function^22^. Beyond their structural and energetic roles, specific amino acids act as metabolic regulators that shape immune cell signalling and phenotype, tightly linking nutrient availability to inflammatory activation^22^. Glutamine serves as a critical anaplerotic substrate, fuelling the tricarboxylic acid (TCA) cycle and supporting nucleotide, amino sugar and glutathione synthesis in rapidly proliferating lymphocytes, neutrophils and macrophages, rendering it conditionally essential during sepsis^23^. Glutamine is needed for T cell proliferation and the production of IL-2 and IFN-γ^24^; it is also required for LPS induction of IL-1 production by macrophages^25^, although it does not appear to be required for M1-like macrophage development^26^. Likewise, arginine metabolism is also altered during sepsis, macrophage activation upregulates inducible nitric oxide synthase (iNOS) to generate nitric oxide (NO) for antimicrobial defense^27^, while arginase-1 (ARG1) competes for the same substrate to support tissue repair and polyamine synthesis^28^. The resulting competition depletes circulating arginine, impairing T cell proliferation and macrophage effector function exacerbating immune dysfunction^28^.

As the response evolves, however, sustained activation pushes immune cells toward functional exhaustion. This transition is accompanied by widespread metabolic collapse. Work from Cheng et al. demonstrated that leukocytes from septic patients with immunoparalysis show broad defects across both glycolysis and OXPHOS, revealing a fundamental failure of cellular energy metabolism^29^. Instead of simply shifting from glycolysis back to OXPHOS, immune cells in late sepsis lose the capacity to engage either pathway effectively. These bioenergetic defects impair antigen presentation, cytokine production, and proliferation, directly linking metabolic failure to immune dysfunction and vulnerability to secondary infection^29^. Thus, immune paralysis is not merely a signalling phenotype but a reflection of profound mitochondrial and glycolytic dysfunction across multiple leukocyte subsets.

These findings align with the broader features of immune exhaustion observed in late sepsis, where macrophages, dendritic cells (DCs) and T cells, exhibit impaired antigen presentation, diminished cytokine production and reduced proliferative capacity^30^. These functional defects are exacerbated by the upregulation of inhibitory receptors such as PD-1 and CTLA-4, which suppress key metabolic and signalling pathways^31^. PD-1 engagement inhibits the PI3K–Akt–mTOR axis, reducing glucose uptake and glycolytic flux while promoting a compensatory shift towards FAO and OXPHOS through AMPK activation^32^. CTLA-4 reinforces this catabolic shift by blocking CD28 co-stimulation, thereby restricting mTOR activation and nutrient transport, limiting energy production and biosynthetic capacity^33^. Together, these checkpoint-mediated pathways compound the oxidative and glycolytic disturbances described above, locking immune cells into a state of energy insufficiency and functional paralysis that drives susceptibility to secondary infection and poor pathogen clearance^34^.

A state of catabolic dominance characterises this late phase. Although initially beneficial in conserving energy and maintaining tissue integrity, persistent catabolism becomes maladaptive, driving muscle proteolysis, nutrient depletion and progressive organ dysfunction^5^. This metabolic collapse, compounded by mitochondrial dysfunction and nutrient depletion, creates a self-reinforcing cycle of energy failure and immune dysfunction. Central to this metabolic reprogramming is the pyruvate dehydrogenase complex (PDC), which links glycolysis to the tricarboxylic acid (TCA) cycle by converting pyruvate into acetyl-CoA^35^. PDC activity is tightly regulated in different immune subsets and conditions. For example, in activated M1 macrophages, PDC activity is inhibited by high PDK1 (pyruvate dehydrogenase kinase 1) expression, promoting aerobic glycolysis to support rapid inflammatory responses^36,37^. In contrast, memory T cells rely on robust PDC activity and OXPHOS for their function and differentiation^38^. In early sepsis, rapid upregulation of PDK1 phosphorylates and inhibits PDC, restricting pyruvate entry into the TCA cycle and reinforcing a glycolytic, pro-inflammatory metabolic profile. This early inhibition supports the acute immune response by sustaining glycolytic ATP production, stabilising HIF-1α through succinate accumulation and promoting effector functions such as cytokine secretion, phagocytosis and NET formation^39^. However, as sepsis progresses into the immunosuppressive phase, persistent PDC inhibition becomes increasingly maladaptive. Immune cells are unable to reactivate OXPHOS or efficiently fuel the TCA cycle, leading to progressive energy failure, impaired antigen presentation, diminished cytokine production, and defective cellular repair^40^. This failure to regain mitochondrial oxidative metabolism is a key driver of late-stage immune exhaustion and functional paralysis, preventing leukocytes from returning to an activation-competent state^40^. In murine models of sepsis, inhibition of PDK1 in monocytes with dichloroacetate (DCA) restored PDC activity, enhanced OXPHOS and improved survival^36^. In contrast, in patients with lactic acidosis, DCA significantly reduced blood lactate but did not change patient outcomes^41^.

Persistent PDC inhibition and the resulting collapse of oxidative metabolism not only limit immune cell function but also exacerbate systemic energy deficits. As the body struggles to meet its metabolic demands in the face of mitochondrial dysfunction, it increasingly turns to alternative nutrient sources, including amino acids, to sustain critical cellular and immune processes^42^. Consequently, sepsis profoundly dysregulates amino acid metabolism, driving the host into a severe hypercatabolic state often described as septic autocannibalism. This state is characterised by rapid skeletal muscle proteolysis to supply amino acids for essential systemic and immune functions^43^.

This proteolysis, primarily mediated by the ubiquitin–proteasome pathway, leads to a net negative nitrogen balance and significant loss of lean body mass, a major contributor to poor outcomes and long-term physical impairment^44^. The liberated amino acids are shuttled to the liver to support critical processes, including hepatic gluconeogenesis to maintain glucose levels in the context of widespread insulin resistance and the accelerated synthesis of acute-phase proteins necessary for the inflammatory response^45^. Beyond these systemic effects, the availability of amino acids also has direct consequences for the immune system, as key substrates such as glutamine, arginine and branched-chain amino acids are required for immune effector function^22^. Depletion of these amino acids can therefore impair adaptive and innate immune responses, contributing to immune paralysis and heightened susceptibility to secondary infections.

**Fig. 2:**
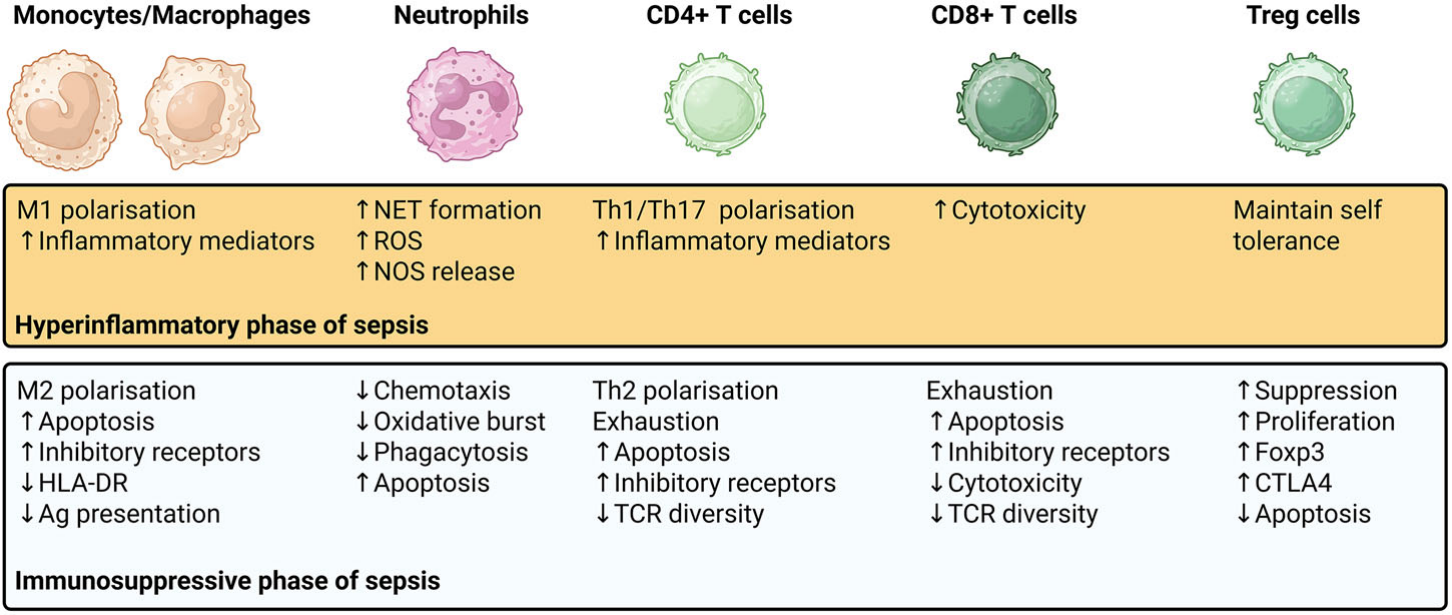
Sepsis-induced changes to immune cells. Sepsis drives profound changes to immune phenotype and function, altering cytokine production and effector capacity. These alterations differ across the hyperinflammatory (upper panel) and immunosuppressive (lower panel) phase of sepsis^104^.

A detailed overview of the impact of metabolic changes on distinct immune cell populations during sepsis is provided in Fig. 2.

## Mitochondrial dysfunction in sepsis

Building on the metabolic reprogramming of immune cells during sepsis mitochondria themselves become central targets of dysfunction with widespread structural and functional impairment precipitating a cellular energy crisis^46^. Mitochondria, enclosed by an outer and inner mitochondrial membrane, contain the electron transport chain (ETC) within the inner membrane. The ETC, comprising complexes I–V, couples electron transfer to proton pumping, generating a proton gradient that establishes the mitochondrial membrane potential essential for ATP synthesis. During sepsis, both the expression and activity of complexes I, III, IV and V have been reported to decline^47^. However, it remains unclear whether these changes directly influence patient prognosis or simply represent secondary effects of acute critical illness^48^.

Beyond energy production, mitochondria regulate reactive oxygen species (ROS), calcium balance, apoptosis, metabolite synthesis and organelle transport. Disruptions in any of these processes compromise cellular homeostasis^49^. In early sepsis, excessive ROS produced by the ETC and NADPH oxidases overwhelms antioxidant defences such as glutathione^50^, damaging mitochondrial lipids, proteins and nucleic acids. Mitophagy, coordinated by PINK1, Parkin, AMPK and sirtuins, is initially upregulated as a protective response removing damaged mitochondria and helping maintain energy homeostasis^46,51^. This process is closely linked to mitochondrial dynamics, as fission is transiently increased in early sepsis, producing smaller mitochondria that can be selectively targeted for mitophagy, while fusion is temporarily suppressed to prioritize organelle quality control^52^. Together, these coordinated changes allow immune cells to cope with acute stress and preserve function^46^.

However, as sepsis progresses, mitophagy becomes impaired or overwhelmed. Damaged mitochondria accumulate, driving a self-amplifying cycle of oxidative stress, ETC dysfunction, reduced ATP production and structural collapse^49^. In late sepsis, persistent fission coupled with impaired fusion and reduced biogenesis, exacerbated by downregulation of the master regulator PGC-1α^48,49^, results in a pool of fragmented, dysfunctional mitochondria^48^. This progressive failure of mitochondrial quality control contributes directly to immune cell exhaustion and the inability to sustain effector function. A key consequence of this mitochondrial deterioration is the release of mitochondrial DNA (mtDNA) that acts as a danger signal, amplifying systemic inflammation via cGAS–STING pathways and triggering cell death^53^. Collectively, these processes exacerbate immune dysfunction and contribute to the multi-organ failure that is characteristic of sepsis^48^.

This progressive mitochondrial dysfunction not only impairs ATP production but also compromises the metabolism of substrates, leading to a collapse of both OXPHOS and FAO^46^. In early sepsis, stress hormones and catecholamines increase lipolysis, releasing free fatty acids that normally undergo mitochondrial β-oxidation via CPT1, the rate-limiting enzyme of FAO^54^. However, ETC dysfunction and downregulation of FAO-related genes via PPARα suppression and NF-κB activation impair mitochondrial FAO, reducing energy generation from fatty acids^54^. In immune cells, FAO supports M2 macrophage polarisation and memory T cell survival, while effector cells rely more on glycolysis. Therefore, early FAO impairment can limit reparative immune responses^55^.

In late sepsis, persistent mitochondrial dysfunction further suppresses FAO, leading to metabolic inflexibility^54^. Immune cells are unable to utilise fatty acids efficiently, contributing to energy collapse and impaired antigen presentation^43^. Accumulation of unoxidised fatty acids may also promote lipid toxicity, exacerbating organ dysfunction^54^. The importance of intact FAO is underscored by experimental models, inhibition of CPT1 markedly increased mortality in a murine bacterial pneumonia model due to defective neutrophil trafficking^56^. Conversely, restoration of FAO through CPT1 activation in models of sepsis-induced organ injury improved mitochondrial function, reduced lipid accumulation and enhanced survival^57^. Recent work has also identified the pattern-recognition receptor TREM2 as an upstream regulator of FAO during sepsis. Elevated TREM2 expression in septic patients correlated with disease severity, and in macrophages, TREM2 suppresses FAO via the SHP1/BTK signalling axis^58^. While genetic deletion of TREM2 in murine sepsis models restored FAO and improved survival^58^, translation remains challenging, as a first-in-human trial of a TREM2 monoclonal antibody (NCT06877533) was recently terminated.

## Emerging immunometabolic biomarkers in sepsis

While organ dysfunction scores and serum lactate remain central to sepsis diagnosis and risk stratification^59–61^, they primarily reflect downstream consequences such as tissue hypoperfusion and organ injury rather than the underlying immunometabolic dysregulation that drives disease heterogeneity. Consequently, there is growing interest in biomarkers that capture the interplay between immune function and metabolism.

Lactate elevation, while a critical warning sign is traditionally viewed as a marker of tissue hypoxia or impaired clearance, which is reflective of the overall systemic collapse^62^. However, lactate functions as a potent immunoregulatory signal^63^. In T cells, lactate can alter metabolism, gene expression, and effector function^64^. It inhibits glycolysis and migration in CD4⁺ and CD8⁺ cells, promotes Th17 differentiation, dampens cytotoxicity, and enhances Treg suppressive activity through GPR81 signalling and Foxp3 upregulation^64^. Lactate serves as an alternative carbon source to support T cell metabolism and polarisation^65^. In macrophages, lactate suppresses pro-inflammatory M1 functions while promoting M2 polarization via HIF-1α, ICER, and GPR81-dependent pathways, reducing cytokine production and inflammasome activity^63,66^. Lactate also exerts epigenetic effects through histone lactylation, a post-translational modification in which lactate-derived lactyl groups are added to lysine residues on histones, in particular H3K18la^67^. Histone lactylation enables transcription of immunosuppressive and reparative genes such as *Nr4a1*, where it facilitates the shift of macrophages towards an M2-like reparative phenotype^67^. Furthermore, high levels of lactylated histone H3K18la have been associated with higher mortality in patients with septic shock^68^. Beyond histone modifications, lactate-derived metabolites, including lactoyl-amino acids, have been identified as potential biomarkers that reflect mitochondrial dysfunction and predict mortality in septic shock^69^. *N*-lactoyl-amino acids when compared to lactate provided greater separation between survivors and non-survivors of septic shock^69^.

Among emerging biomarkers in sepsis, CD36 has gained particular interest due to its mechanistic links to immunometabolic dysfunction^70^. Studies using CD36-deficient mice in sepsis models showed reduced inflammation and improved survival, underscoring a role for CD36 in exacerbating inflammation^71^. CD36, a fatty acid translocase and scavenger receptor, facilitates high-affinity uptake of long-chain fatty acids and its upregulation during sepsis promotes intracellular lipid accumulation and lipotoxicity^72^. CD36 expression increased on macrophages during sepsis, reflecting a shift toward lipid-dependent metabolism contributing to the inflammatory response and organ damage. This metabolic overload contributes to impaired FAO enhancing mitochondrial dysfunction^70^. These changes are further compounded by sepsis-associated suppression of the sirtuins SIRT1 and SIRT3, key NAD⁺-dependent regulators of mitochondrial biogenesis and FAO^73^. Reduced sirtuin activity diminishes mitochondrial integrity amplifying organ injury^73^. Highlighting its clinical relevance, CD36 reflects these metabolic derangements and may indicate both disease severity and the risk of organ dysfunction in sepsis^74^.

The SLC27 family of fatty acid transport proteins (FATPs) regulate lipid metabolism in immune cells and their dysregulation has been implicated in sepsis making them potential biomarkers^75^. Six isoforms mediate long-chain fatty acid uptake and conversion into acyl-CoAs to support cellular and immune function^76^. Different immune cells express distinct forms, myeloid-derived suppressor cells upregulate SLC27A3, SLC27A4 and SLC27A6^77^, while SLC27A1 is expressed in CD8⁺ T cells to support their effector functions^78^. SLC27A2, expressed in myeloid cells, is upregulated in sepsis, driving excessive fatty acid influx, mitochondrial dysfunction and metabolic stress. This contributes to early inflammation and late immunosuppression, as SLC27A2 activity promotes production of immunosuppressive lipid mediators^79^.

SLC2A1 (GLUT1) is a central regulator of immunometabolic reprogramming in sepsis and a promising biomarker of disease severity. Its expression is rapidly upregulated by pathogen-associated signals and inflammatory cytokines, while elevated SLC2A1 levels in septic patients correlate with heightened immune activation^80^. SLC2A1 also contributes to the resolution of infection. In MRSA-induced skin and soft-tissue infection, loss of myeloid SLC2A1 impaired NADPH generation and PPARγ activation delaying resolution^81^.

Finally, the amino acid transporter SLC7A5 is increasingly recognised as relevant in sepsis^82^, where it supports immune cell activation especially for Th1 and Th17 cells by importing large neutral amino acids, such as leucine, phenylalanine, tryptophan and the immunomodulatory metabolite kynurenine^83^. The influence of SLC7A5 on T cell fate decisions depend largely on mTORC1 signalling^84^. Sepsis leads to the accelerated depletion of tryptophan and subsequent kynurenine accumulation, making SLC7A5 a key regulator of the kynurenine pathway and its downstream effects on immune tolerance and T cell suppression through the promotion of Treg activity^85^. Altered SLC7A5 expression reflects shifts in immune metabolic demand, with upregulation marking late immunosuppression.

Together, these potential biomarkers demonstrate that immunometabolic alterations are central to the pathophysiology of sepsis (Table 1). By complementing traditional markers of organ dysfunction, they may offer a more mechanistic understanding of disease progression and severity.

**Table 1:** Immunometabolic biomarkers of sepsis

Biomarker	Pathway	Clinical Relevance
Serum lactate	Marker of aerobic and anaerobic glycolysis; immunometabolic signalling via GPR81	Differentiates sepsis from septic shock; dynamic levels correlate with severity; contributes to T cell and macrophage immunosuppression^64,98^
Lactate-derived modifications (lactoyl amino acids/ histone lactylation)	Markers of mitochondrial dysfunction. Influence amino acids and histone lactylation, changing gene expression	Linked to mortality in septic shock; more nuanced metabolic readout than static lactate^99^
Amino acids (Glutamine)	Supports mTOR signalling, modulates NF-κB and MAPK pathways; fuels immune cells via TCA anaplerosis	Associated with increased severity and mortality in early sepsis; influences immune cell responsiveness^23,24^
Amino acids (tryptophan/kynurenine, arginine)	IDO1-mediated tryptophan catabolism; NAD + /NADH imbalance	Reflects immune suppression, oxidative stress and poor prognosis; kynurenine/tryptophan ratio predicts T cell exhaustion^83,100^
Free fatty acids	Increased lipolysis and impaired fatty acid oxidation; mitochondrial dysfunction	Correlates with clinical score, marker of mitochondrial failure^101^
Acylcarnitines	Impaired fatty acid oxidation; mitochondrial dysfunction	Correlates with severity and mortality, marker of mitochondrial failure^100,102^
Glucose transporters (SLC2A1)	Glucose uptake transporter regulating glycolysis and immune activation in early sepsis	Upregulated in activated immune cells; marks shift toward aerobic glycolysis; associated with inflammatory activation and severity^81^
Long-chain fatty acid transporters (SLC27)	Facilitates uptake of long-chain fatty acids for FAO and membrane synthesis	Altered expression indicates dysregulated lipid utilisation in sepsis; reflects impaired energy generation and immune dysfunction^78,79^
CD36 scavenger receptor	Fatty acid translocase mediating uptake of long-chain fatty acids, oxidised lipids; integrates lipid metabolism with innate immune signalling	Reflects dysregulated lipid handling in early sepsis and contributes to inflammatory activation, impaired phagocytosis; associated with disease severity and organ dysfunction^71,72^
Amino acid transporters (SLC7)	Controls uptake of neutral amino acids; regulates mTOR activation and redox balance	Dysregulation affects T cell activation, cytokine responses; associated with severity of metabolic and immune dysfunction in sepsis^83,84^

## Avenues for monitoring immunometabolism in sepsis

New technologies are increasingly enabling real-time and high-resolution monitoring of immunometabolism in sepsis, offering the potential for more precise assessment of immune dysfunction. Advances in single-cell multi-omics now allow simultaneous profiling of gene expression, surface phenotype and metabolic state, revealing immunometabolic rewiring. A combined transcriptome–metabolome analysis identified 40 gene–metabolite pairs unique to sepsis compared with SIRS, many linked to macrophage metabolic pathways, enabling discrimination between pathogen-driven and sterile inflammation^86^. Several computational frameworks now extend single-cell omics by inferring metabolic flux directly from transcriptomic data. Tools such as METAFlux^87^, scFEA and its FLUXestimator interface^87^, together with Compass^88^ use network-based or machine-learning approaches to predict pathway activity, metabolite accumulation, and metabolic switching at single-cell resolution. These methods enable reconstruction of glycolytic, fatty-acid–driven or oxidative programs without requiring direct metabolite measurements, providing a powerful complement to experimental single-cell datasets.

Building on these advances, flow cytometric approaches are now directly incorporating metabolic analysis allowing for a deeper mapping of immune dysfunction. Techniques such as such as scMEP, a mass cytometry (CyTOF)-based method^89^ or MET-Flow spectral cytometry^90^ enable the quantification of rate-limiting enzymes, nutrient transporters and transcription factors, serving as proxies for distinct metabolic pathways. Both approaches targeting enzymes involved in major metabolic pathways including hexokinase 1 (HK1), PFK2 and SLC2A1 for glycolysis, isocitrate dehydrogenase 2 (IDH2) for the TCA cycle, G6PD for the pentose phosphate pathway and CPT1A for FAO. They also include markers of mitochondrial mass and metabolic signalling^89,90^. Importantly, these panels are adaptable, allowing them to be tailored to be more species-relevant or specific to the particular intermediary metabolic processes that are under investigation in the context of sepsis. Flow cytometric techniques such as SCENITH can also be incorporated, as this method allows for functional measurements of metabolic dependencies alongside immune phenotypes^91^. This methodology quantifies cellular metabolic activity by assessing alterations in protein synthesis rates in response to targeted metabolic inhibitors. It uses the incorporation of puromycin as a rapid, quantitative readout for the overall translational rate, which serves as a surrogate marker for the underlying energetic state^91^. Recent work extends its application to sepsis showing that Tregs in critically ill patients acquire an increased glycolytic phenotype that helps stabilise suppressive markers that correlate with the severity of the illness^92^.

Complementing this, fluxometry techniques allow for real-time assessment of cellular metabolic function^93^, thereby tracking dynamic changes in immune cell bioenergetics during sepsis. Indeed, Seahorse technology has been used to show that sepsis induces persistent metabolic reprogramming in CD4⁺ T cells. Even after recovery, these cells failed to reset their metabolic state maintaining elevated glycolysis and oxygen consumption upon activation, indicative of long-term immune dysregulation^15^. However, while these techniques offer powerful research insights into cellular metabolism in sepsis, their usefulness in a clinical setting is limited by their ex vivo nature, high cost and technical complexity, which prevent rapid, real-time patient monitoring. The continuous refinement of these methodologies, particularly the development of miniaturised or small-volume point-of-care techniques that incorporate metabolism, will ultimately lead to bedside monitoring which could transform the clinical management of sepsis^94^.

## Conclusions

Integrating immunometabolic monitoring into clinical practice offers a novel way to track the trajectory of host immune dysfunction in sepsis. Serial assessment of immunometabolic shifts can reveal changes in fuel utilisation that mirror transitions between hyper-inflammation and immunosuppression, effectively functioning as a signature of immune bioenergetics, information not fully captured by clinical scores^59^ or measures of serum lactate^95^. Longitudinal profiling could contextualise bedside changes with underlying cellular respiration, glycolysis and metabolic flexibility.

Immunometabolic assays could also serve as immediate pharmacodynamic readouts for patient-directed clinical trials, confirming target engagement and helping stratify responders. Fluxometry has demonstrated that restoring pyruvate dehydrogenase flux with dichloroacetate improved oxidative capacity and improved survival in preclinical sepsis models^96,97^. While interventions targeting TREM2-high myeloid states were tracked using FAO-linked metrics^58^. However, adoption would require rigorous standardisation.

With validated methodologies and integration into existing endotyping frameworks, immunometabolic monitoring can translate fundamental biology into actionable clinical insights. By identifying dysfunction before irreversible organ injury, it offers a path to guide metabolically targeted therapies and provide patient-specific metrics that complement current scoring systems.

## Data Availability

No datasets were generated or analysed during the current study.
